# A Fallacious Theory Exploded

**Published:** 1879-06

**Authors:** 


### A FALLACIOUS THEORY EXPLODED,

Mankind has a strikingly developed tendency to theorize. In the untiring advance of scientific investigation, when facts are wanting to satisfactorily account for any natural phenomenon some genius steps to the front with a nicely constructed theory to supply the want of actual knowledge; and after a little discussion it is either accepted or rejected by the scientific world. When a theory has been once adopted, however erroneous it may be, it is then taught as a proven fact, and investigation on the part of scientists in general ceases. There are minds whose activity can never be lulled to sleep by any theory, however plausible, and through these, at varying intervals, burst upon the world startling and important discoveries. There is no science that has not, within a comparatively recent period, undergone a revolution in consequence of a single discovery, the offspring of a single active, untiring mind. The undulatory theory of sound has long been received as established, but lately has arisen an investigator who has, to the minds of many, completely overthrown the doctrine. He boldly attacks the whole scientific world, and thus far his position has only been strengthened by the investigations and criticisms of scientific men. We have before us his controversy with Prof. R. L. Brockett, on the subject, which is the most interesting scientific discussion we have ever read, and in which the learned professor and the popular theory are roughly handled. The same author (who disguises himself under the *non de plume* of "Wilford") in another work, "Evolution Evolved," arrays himself against Darwin and evolutionists in general. It is said by all who have had the opportunity of examining the work, that every position assumed is sustained by uncontrovertible arguments. It is a work that should be in the hands of every clergyman, physician and all other persons who are interested in unravelling the mysteries of human life and scientific research in general. The Brockett-Wilford discussion is mailed free by the publishers to all who send their address for that purpose. The publishers are Hall & Co., 234 Broadway, New York.

